# Educational mobility and weight gain over 13 years in a longitudinal study of young women

**DOI:** 10.1186/1471-2458-14-1219

**Published:** 2014-11-25

**Authors:** Natalie Holowko, Mark Jones, Leigh Tooth, Ilona Koupil, Gita Mishra

**Affiliations:** Centre for Longitudinal and Life Course Research, School of Population Health, University of Queensland, Herston Road, 4006 Brisbane, Australia; Centre for Health Equity Studies (CHESS), Stockholm University/Karolinska Institute, Sveavägen 160, 10691 Stockholm, Sweden

**Keywords:** Educational status, Longitudinal studies, Social inequalities, Weight trajectory, Weight gain

## Abstract

**Background:**

Limited evidence exists about the role of education and own educational mobility on body weight trajectory. A better understanding of how education influences long term weight gain can help us to design more effective health policies.

**Methods:**

Using random effects models, the association between i) highest education (n = 10 018) and ii) educational mobility over a 9 year period (n = 9 907) and weight gain was analysed using five waves of data (over 13 years) from the Australian Longitudinal Study on Women’s Health 1973–78 cohort (from 18–23 years to 31–36 years).

**Results:**

Highest educational attainment was inversely associated with weight at baseline and weight gain over 13 years. Compared to high educated women, those with a low (12 years or less) or intermediate (trade/certificate/diploma) education, respectively, weighed an additional 2.6 kg (95% CI:1.9 to 3.1) and 2.5 kg (95% CI:1.9 to 3.3) at baseline and gained an additional 3.9 kg (95% CI:2.6 to 5.2) and 3.1 kg (95% CI:2.6 to 3.9) over 13 years. Compared to women who remained with a low education, women with the greatest educational mobility had similar baseline weight to the women who already had a high education at baseline (2.7 kg lighter (95% CI:-3.7 to -1.8) and 2.7 kg lighter (95% CI:-3.4 to -1.9), respectively) and similarly favourable weight gain (gaining 3.1 kg less (95% CI:-4.0 to -2.21) and 4.2 kg less (95% CI:-4.8 to -3.4) over the 13 years, respectively).

**Conclusions:**

While educational attainment by mid-thirties was positively associated with better weight management, women’s weight was already different in young adult age, before their highest education was achieved. These findings highlight a potential role of early life factors and personality traits which may influence both education and weight outcomes.

**Electronic supplementary material:**

The online version of this article (doi:10.1186/1471-2458-14-1219) contains supplementary material, which is available to authorized users.

## Background

Overweight and obesity are of high concern due to adverse health risks associated with increased body weight; including cardiovascular disease, hypertension and cancer [1]; excess obstetric risks for both mother and offspring [2]; and the intergenerational transfer of adult metabolic risk through intrauterine growth and prenatal programming of adipose tissue [3]. With a noted steady pattern of weight gain across the life course [4, 5], investigating determinants of patterns of long term weight change is of increasing priority.

Studies in high income countries, including the U.S., Australia, U.K. and Sweden, show an inverse association between adult socioeconomic position (SEP) and body mass index (BMI) across the life course, using education [4–10] and occupation [4, 8, 11]. This trend is found among women in early-mid [6, 7, 9, 10, 12–14] and mid-late adulthood [7, 8, 11], while some studies report no association [15, 16]. Suggested mechanisms include health related behaviours and social/material resources with an established association with weight gain; including physical activity (low levels), high fat/energy intake [8, 17]; smoking (quitting) [8]; alcohol consumption (mixed associations, potentially u-shaped) [18]; sitting time (increased levels) [19]; marital status (partnered), higher initial BMI and having children [5].

High mean weight gain over four [15] and 10 year [5] periods is found in women aged 18–23 years, and over five years [20] in women aged 35–44 years; with weight differences by SEP increasing among younger female cohorts [14, 15]. This trend is significant in itself, let alone increasing maternal BMI being considered a high risk obstetric condition, associated with gestational diabetes mellitus [2] and hypertension [21], as well as offspring obesity in childhood [22].

Previous research using the Australian Longitudinal Study on Women’s Health (ALSWH) 1973–78 cohort found that highly educated women in their early twenties had a significantly lower BMI four years later [23]; and that in relation to having children, high educated women (at age 28–33 years) also gained relatively less weight over a 10 year period [5]. Within the U.S. Coronary Artery and Risk Development in Young Adults (CARDIA) study [24], among 18–30 year old women, only education at follow up seven years later was inversely associated with BMI change.

Few studies have explored the effect of when education is measured (early or later adulthood) and changes in education level on body weight trajectory. Such knowledge may assist in better understanding the social patterning of body weight and differing associations found between different studies [15, 16, 23, 24].

This research explores how education may influence long term weight gain in women, by investigating the effect of education measured in early and later adulthood. We focused on detailed investigation of the relationship between i) highest achieved education by mid-thirties and ii) educational mobility (from early-mid twenties to early-mid thirties) with baseline body weight and rate of change over 13 years. We also explored baseline characteristics in women based on their highest achieved education.

## Methods

### Study participants

The ALSWH began in 1996, using a sample of women randomly selected from the national health insurance database (Medicare), consisting of all Australian citizens and residents, with a deliberate oversampling of rural/remote women. Detailed information about the three original ALSWH cohorts (41 500 women) can be found elsewhere [25]. The ALSWH study is approved by the Human Research Ethics Committees of the Universities of Newcastle and Queensland. Informed consent was given by all participants of the study.

Our sample was drawn from the cohort born 1973–78; aged 18–23 at baseline and found generally representative of the female population for their age [26].

Of the 14 247 women who answered the baseline survey, 9 688 (68%) completed Survey Two (2000; aged 22–27 years); 9 081 (64%) completed Survey Three (2003; aged 25–30 years); 9 145 (64%) completed Survey Four (2006; aged 28–33 years); and 8 200 (58%) completed Survey Five (2009; aged 31–36 years). Relatively high attrition between baseline and Survey Two is thought to result from, among other things, a high level of geographical mobility and changes in surname upon marrying [25].

Our sample was restricted to women with body weight reported in two or more surveys, resulting in 11 436 women (see Additional file 1). For our analysis with a main exposure of highest achieved education the sample size was 10 018 women, and for educational mobility (from early to mid-twenties up to early to mid-thirties) it was 9 907 women.

We additionally ran the analyses for both exposures using data imputed for all women with one body weight (n = 13 862). We used PROC MI, with 20 imputations using fully conditional specification, to impute all outcomes, exposures, and covariates used in the mixed models. We also included auxiliary variables associated with missingness in the imputation model [27].

### Measures

### Outcome - Body weight and weight gain

At each survey, women were asked “How much do you weight without clothes or shoes (if you are not sure, please estimate)”. Women could answer in kilograms/grams or stones/pounds (these measurements were then converted into kilograms/grams). From Survey Four (2006, aged 28–33 years) onwards, pregnant women were specifically asked to report their weight in the month prior to their pregnancy. Given this, weight for women pregnant at Survey One (1996, n = 90), Two (2000, n = 78) or Three (2003, n = 30) was excluded from that respective survey.

### Exposures - Indicators of socioeconomic position

Two key SEP indicators were explored: (i) highest achieved education, measured as participants own education at Survey Five (or Survey Four if missing) categorised as: low (high school certificate or lower), intermediate (trade/apprentice/certificate/diploma) or high (degree/higher degree); and (ii) educational mobility, from Survey Two (carried forward from Survey One if missing) to Survey Five (carried forward from Survey Four if missing) (see Additional file 1). Using education from Survey Two gave the youngest women in the cohort opportunity to have completed a degree. Educational mobility was categorised as: stable low, low-intermediate, stable intermediate, upgrade to high (low-high and intermediate-high) and stable high.

Sensitivity analyses for education level were conducted using i) education at Survey Five only (n = 9 037); and ii) educational mobility from Survey Two to Five (no carrying forward) as the main exposure (n = 8 162) (results available upon request). Sensitivity analyses using all women with one body weight are presented in Additional files 2 and 3.

### Covariates

#### Demographic, psychosocial, material, behavioural and reproductive variables

Due to deliberate initial oversampling of women living in rural and remote areas of Australia, area of residence was adjusted for in all models, categorised as urban (major cities), rural (inner regional), and remote (outer regional/remote). Additionally, all models included age and height centred at the cohort means of 20.8 years and 165.9 cm. The following variables commonly associated with socioeconomic position and body weight were considered for inclusion.

At each survey, questions were asked to determine marital status (married/defacto, separated/divorced/widowed, never married); living arrangement (partner/children; alone; parents/relatives; non-family); number of children (none; one; two; three or more) based on reported dates of birth of children; smoking status (current smoker; non-smoker; ex-smoker); alcohol intake (never/rarely; risky/high risk 15+ drinks/week; low risk ≤14 drinks/week; never/rarely), based on the Australian National Health and Medical Research Council (NHMRC) guidelines [28]; physical activity as MET/mins per week (nil/sedentary 0–40; low 40- < 600; moderate 600 - < 1200; high ≥1200) [29]; ability to manage on income (impossible/always difficult; sometimes difficult; not too bad/easy) ; body shape dissatisfaction (not at all; slightly; moderately; markedly); self-rated health (excellent; very good; good; poor/fair); and health transition (better; about the same; worse), comparing health to a year ago.

Mental health (poor ≤52; good >52) was measured using the Mental Health Index (MHI-5) subscale of the SF-36 (Medical Outcomes Study short form 36 health survey) [30]. Age at birth of first child was based on most recent information. Country of birth was asked at baseline (Australia; other English speaking; Europe (including Turkey, Russia); Asia; other (including the Middle East)).

For descriptive analyses, the World Health Organisation’s (WHO) categories for BMI were used; underweight (<18.50 kg/m^2^), healthy weight (18.50-24.99 kg/m^2^), overweight (25.00-29.99 kg/m^2^) and obese (≥30.00 kg/m^2^) [31].

### Statistical analyses

Cross sectional analyses investigated trends in weight with increasing age, from 18–23 to 31–36 years. Unweighted statistics are presented, since weighting for area of residence (due to an oversampling of rural women) did not result in significantly different results.

Random effects models (using SAS PROC MIXED) were used to investigate the association of education and educational mobility with weight measured at five time points over 13 years. While mixed models are robust to missing data under the assumption of missing at random (MAR), results from sensitivity analyses using imputed data can be found in Additional files 2 and 3. Each subject had their own intercept and slope (random effects), accounting for correlations between observations within individuals [32]. All other variables were modelled as fixed effects. The time scale used was number of years between baseline (1996) and the return of each survey. A quadratic term for time was included in all models, given a slight attenuation in the increase of weight over time. Results from the random effects Models 1 were used to plot baseline weight and weight gain overtime in the figures presented.

Final selection of covariates was based on 10% or greater change in primary point estimates, which according to Greenland [33] is more robust than stepwise regression or significance testing approaches. None of the covariates were highly correlated with the SEP exposures, which could have introduced bias in the adjusted models. Covariates were included as categorical or ordinal, fixed or time-varying using model comparison. Model assessment was made comparing Akaike Information Criteria (AIC) goodness of fit statistics, with lower values indicating a better fit. All analyses were completed in SAS version 9.4 for Windows (SAS Institute Inc., Cary, NC).

## Results

A higher percentage of the 2 811 women excluded from our final sample had a low education, no children, never/rarely drink alcohol, had poor mental health and were underweight, born outside of Australia, current smokers and sedentary (see Table 1). These women had a younger mean age at birth of first child.

**Table 1:** **Baseline characteristics of 1973–78 cohort ALSWH women included/excluded from the sample** ^**†**^ **(N = 14 247)**

	Included N = 11,436 ^†^	Excluded N = 2,811 ^†^	P-value*
**Baseline characteristics**			
*Mean (Std Dev)*			
**Weight** (kg)	62.7 (12.5)	61.8 (13.3)	<0.0001
**Height** (cm)	165.9 (7.1)	165.3 (8.2)	<0.0001
Age at birth of first child**	27.1 (4.3)	23.1 (3.7)	0.0089
Percentage (%)			
**BMI**			<0.0001
Underweight (<18.5)	9.5	13.6	
Normal weight (18.5 -24.9)	69.0	64.3	
Overweight (25.0 – 29.9)	15.3	15.4	
Obese (≥30.0)	6.2	6.7	
Education***			<0.0001
Low	70.7	74.0	
Intermediate	17.1	19.7	
High	12.2	6.3	
**Number of children**			<0.0001
No children	93.1	97.6	
1	5.3	1.6	
2	1.3	0.7	
3+	0.3	0.1	
**Marital status**			<0.0001
Never married	77.7	71.8	
Married/de facto	21.6	26.4	
Separated/divorced/widowed	0.7	1.9	
**Living arrangement**			<0.0001
Parents/relatives	49.3	44.9	
Partner/children	26.9	35.9	
Non-family	17.7	13.1	
Alone	6.1	6.1	
**Physical activity**			<0.0001
Nil/Sedentary	6.0	10.0	
Low	37.1	37.3	
Moderate	13.4	11.7	
High	43.5	41.0	
**Alcohol intake**			<0.0001
Never/rarely	41.8	49.8	
Low risk	52.9	43.5	
Risky/high risk	5.3	6.7	
**Smoking status**			<0.0001
Non-smoker	54.1	44.9	
Ex-Smoker	15.1	15.9	
Current smoker	30.8	39.2	
**Mental health (based on MHI-5)**			<0.0001
Poor (≤52)	20.7	25.8	
Good (>52)	79.3	74.2	
**Self-rated health**			<0.0001
Excellent	12.8	11.4	
Very good	39.9	33.1	
Good	35.8	40.7	
Poor/Fair	11.5	14.8	
**Ability to manage on income**			<0.0001
Easy/Not too bad	50.1	41.4	
Difficult sometimes	32.6	35.5	
Impossible/Always difficult	17.4	23.1	
**Body shape dissatisfaction**			<0.0001
Not at all	8.8	12.8	
Slightly	25.9	23.4	
Moderately	31.9	28.9	
Markedly	33.3	34.9	
**Country of birth**			<0.0001
Australia	92.6	86.9	
Other English speaking	3.6	4.6	
Europe	0.9	1.6	
Asia	2.0	5.4	
Other (incl. Middle East)	0.8	1.5	
**Area of residence**			0.4444
Urban (major cities)	51.9	51.4	
Rural (inner regional)	30.4	29.8	
Remote (outer regional/ remote)	17.7	18.8	

^†^sample sizes change slightly due to missing values for some variables.

*P-values from independent t-tests for continuous variables and from Pearson chi square tests for categorical variables.

**Age at birth of first child is based on reported information up to Survey Five.

***Education at baseline (Low - higher school certificate or lower (≤12 years), Intermediate - trade/certificate/diploma, High - degree/higher degree).

Mean body weight increased from 62.7 kg at Survey One to 71.3 kg at Survey Five. The greatest increases in educational mobility were between baseline and Survey Two; at which point 35% of women had a low, 26% had an intermediate and 39% a high education (results available upon request). In contrast, at Survey Five 18% of women had a low, 28% an intermediate and 54% a high education.

Results from the random effects model show an inverse association between highest achieved education and both baseline weight and weight gain. Compared to women with a high education (see Table 2, model 1) who were lightest at baseline and gained the least per year (~0.8 kg), women with a lower education were approximately 2.5 kg heavier at baseline and gained approximately an additional 0.24-0.29 kg/year.

**Figure 1:**
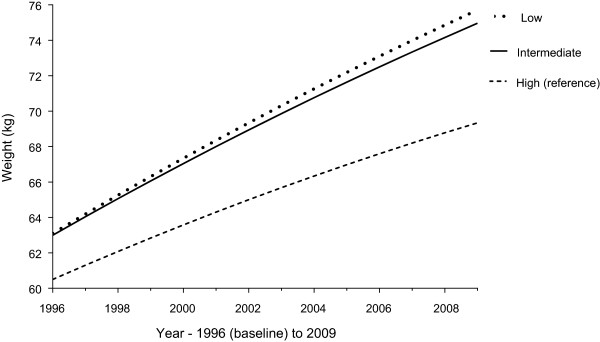
**Highest achieved education and weight gain over 13 years in women from the 1973–78 ALSWH cohort (n = 9 573).** Difference in baseline weight and weight gain over 13 years (random effects model with a random intercept and slope, adjusted for area of residence and baseline centred age and height), based on highest achieved education at Survey Five (age 31–36 years). Education categorised as ‘Low’ (higher school certificate or lower - ≤12 years), ‘Intermediate’ (trade/certificate/diploma) or ‘High’ (degree or higher).

Weight gain among all education groups has only slightly attenuated over time (see Figure 1).

**Table 2:** **Baseline weight and weight gain** ^*^ **over 13 years by highest education** ^**†**^ in 1973–78 cohort ALSWH women (n = 9 573**)

	% weighted (unweighted)	Model 1 Estimate (95% CI)	Model 2 Estimate (95% CI)
**Baseline weight (kg)**		60.51 (60.06, 60.97)	58.89 (58.11, 59.68)
*Difference in baseline weight by highest achieved education* ^†^			
High	51.3 (46.9)	Reference	Reference
Intermediate	29.5 (31.1)	2.48 (1.87, 3.08)	1.67 (1.08, 2.26)
Low	19.3 (22.0)	2.63 (1.93, 3.33)	1.70 (1.00, 2.39)
**Increase per year (kg)**		0.82 (0.77, 0.87)	1.18 (1.12, 1.24)
*Difference in increase per year by highest achieved education* ^†^			
High		Reference	Reference
Intermediate		0.24 (0.19, 0.28)	0.23 (0.19, 0.28)
Low		0.29 (0.24, 0.35)	0.27 (0.22, 0.33)
Attenuation per year (time*time)		-0.02 (-0.26,-0.20)	-0.05 (-0.06, -0.05)

^*^Random effects models (intercept and slope) with weight measured at age 18–23 years, 22–27 years, 25–30 years, 28–33 years and 31–36 years.

^†^Education achieved at Survey Five (Low - higher school certificate or lower (≤12 years), Intermediate - trade/certificate/diploma, High - degree/higher degree).

**Sample slightly smaller than the 10,018 women who had a value for highest achieved education, due to missing values for some covariates.

Model 1 – Baseline centred age, baseline centred height and area of residence.

Model 2- Model 1 + country of birth, physical activity, alcohol intake, mental health, income management, self-rated health, age at first birth, living arrangements, marital status, shape dissatisfaction.

### Education and weight gain over 13 years

The significant association between highest achieved education and both baseline weight and weight gain remained after adjusting for covariates; social differences in weight gain did not change, while differences in baseline weight by education level narrowed slightly (see Table 2, model 2).

### Educational mobility and weight gain over 13 years

Results from the random effects model show weight at baseline was significantly different for women defined by their educational mobility. Compared to women with a stable low education, who were the heaviest at baseline (see Table 3, model 1), women with a stable high education or who upgraded to a high education (greatest educational mobility) weighed significantly less at baseline (~2.7 kg lighter; 1.8 and 2.1 kg lighter, respectively, when fully adjusted).

**Figure 2:**
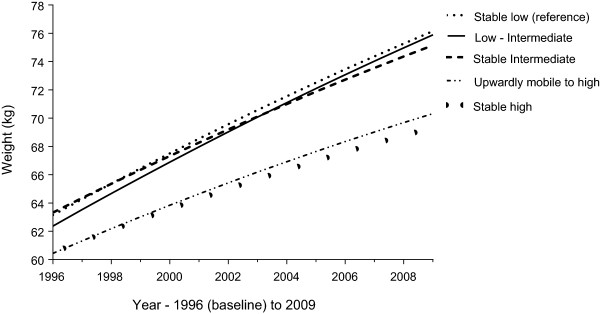
**Educational mobility and weight gain over 13 years in women from the 1973–78 ALSWH cohort (n = 9 463).** Difference in baseline weight and weight gain over 13 years (random effects model with a random intercept and slope, adjusted for area of residence and baseline centred age and height), based on educational mobility from Survey Two (age 22–27 years) to Survey Five (age 31–36 years). Educational mobility categorised as ‘stable low’ (low-low), ‘low-intermediate’, ‘stable intermediate’ (intermediate-intermediate), ‘upgrade to high education’ (low-high or intermediate-high) or ‘stable high’ (high).

Highest achieved education was indicative of weight change, with women who achieved the highest education level by Survey Five gaining slightly less weight per year. Compared to women with a stable low education who gained 1.1 kg/year (~1.5 kg/year fully adjusted), women with a stable high education gained 0.3 kg/year less and women who upgraded to the highest education gained 0.24 kg/year less (0.2 kg/year fully adjusted). There was no significant difference between the women with a stable low and low-intermediate education. Women with a stable intermediate education gained marginally less per survey compared to women who were stable low. Weight gain among all categories of educational mobility has slightly attenuated over time (see Figure 2).

**Table 3:** **Baseline weight and weight gain** ^*^ **over 13 years by educational mobility** ^**†**^ in 1973–78 cohort ALSWH women (n = 9 463**)

	% weighted (unweighted)	Model 1 estimate (95% CI)	Model 2 estimate (95% CI)
**Baseline weight (kg)**		63.15 (62.46, 63.85)	57.85 (56.77, 58.92)
*Difference in baseline weight by educational mobility* ^†^			
Stable low	18.9 (21.7)	Reference	Reference
Low-intermediate	9.9 (10.9)	-0.78 (-1.8, 0.27)	-0.78 (-1.78, 0.21)
Stable intermediate	19.8 (20.5)	0.17 (-0.68, 1.02)	0.31 (-0.51, 1.12)
Upgrade to high	12.8 (12.0)	-2.71 (-3.68, -1.75)	-2.13 (-3.07, -1.19)
Stable high	38.6 (34.9)	-2.66 (-3.41, -1.91)	-1.77 (-2.52, -1.02)
**Increase per year (kg)**		1.12 (1.06, 1.18)	1.45 (1.39, 1.52)
*Difference in increase per year by educational mobility* ^†^			
Stable low		Reference	Reference
Low-intermediate		0.04 (-0.09, 0.07)	0.01 (-0.07, 0.08)
Stable intermediate		-0.09 (-0.15, -0.22)	-0.07 (-0.13, -0.002)
Upgrade to high		-0.24 (-0.31, -0.17)	-0.20 (-0.27, -0.13)
Stable high		-0.32 (-0.37, -0.26)	-0.28 (-0.33, -0.22)
Attenuation per year (time x time)		-0.02 (-0.03, -0.02)	-0.05 (-0.06, -0.05)

^*^Random effects models (intercept and slope) with weight measured at age 18–23 years, 22–27 years, 25–30 years, 28–33 years and 31–36 years

^†^Change in education level from age 22–27 years to age 31–36 years: (Low - higher school certificate or lower (≤12 years), Intermediate - trade/certificate/diploma, High - degree/higher degree). Upgrade to high includes women who had a low (70%) or intermediate (30%) education who later upgraded to a high education.

**Sample slightly smaller than the 9,907 women who had a value for change in education level, due to missing values for some covariates.

Model 1 – baseline centred age, baseline centred height and area of residence.

Model 2 - Model 1 + physical activity, alcohol intake, mental health, self-rated health, number of children, smoking, age at first birth, living arrangement, marital status, health transition, shape dissatisfaction, income management and country of birth.

Sensitivity analyses for both exposures (see Methods) showed similar associations to those presented with marginally lower estimates (results available upon request). Additionally, sensitivity analyses using imputed data showed the same associations to those presented (see Additional files 2 and 3).

### Baseline characteristics of women based on their highest achieved education

While the mean age at baseline was similar for all education groups, women with a high education by Survey Five were significantly lighter and taller at baseline (see Table 4). At baseline, a greater proportion of these women had never had children (98%); with an older mean age at birth of first child (29.1 years), compared to intermediate (26.6 years) and low (25.3 years) educated women.

**Table 4:** **Baseline characteristics in 1973–78 cohort ALSWH women based on highest achieved education** ^**†**^ **(n = 10 018)**

	Highest achieved education ^†^			
	Low (21%) n = 2,087 ^‡^	Intermediate (30%) n = 2,994 ^‡^	High (49%) n = 4,937 ^‡^	chi ^2^ /F statistic (P-value)
***Baseline characteristics***				
*Mean (Std Dev)*				
***Age*** *(years)*	20.8 (1.5)	20.8 (1.4)	20.7 (1.5)	9.23 (<0.0001)
***Weight*** *(kg)*	63.7 (13.1)	63.6 (13.3)	61.8 (11.1)	23.6 (<0.0001)
***Height*** *(cm)*	165.4 (7.4)	165.7 (7.3)	166.4 (6.8)	18.4 (<0.0001)
Age at birth of first child**	25.3 (4.3)	26.6 (4.1)	29.1 (3.4)	529.6 (<0.0001)
Percentage (%)				
**BMI**				* 44.3 (<0.0001)
Underweight (<18.5)	9.2	9.1	9.4	
Normal weight (18.5 -24.9)	62.3	66.4	73.5	
Overweight (25.0 – 29.9)	18.9	16.5	13.3	
Obese ( ≥30.0)	9.6	8.0	3.8	
**Number of children**				509.9 (<0.0001)
No children	83.7	91.3	98.2	
1	12.3	6.8	1.5	
2	3.3	1.6	0.2	
3+	0.7	0.3	0.02	
**Marital status**				704.0 (<0.0001)
Never married	62.3	71.5	88.7	
Married/de facto	36.0	27.7	11.1	
Separated/divorced/widowed	1.7	0.8	0.2	
**Physical activity**				73.5 (<0.0001)
Nil/Sedentary	8.4	6.6	4.4	
Low	40.1	37.6	35.4	
Moderate	12.6	13.4	13.9	
High	38.9	42.4	46.3	
**Alcohol intake**				121.5 (<0.0001)
Never/rarely	48.8	42.8	36.9	
Low risk	44.8	51.6	58.7	
Risky/high risk	6.4	5.6	4.4	
**Smoking status**				429.7 (<0.0001)
Non-smoker	40.9	47.8	65.2	
Ex-Smoker	18.4	17.0	12.7	
Current smoker	40.7	35.2	22.1	
**Mental health (based on MHI-5)**				56.8 (<0.0001)
Poor (≤52)	24.4	21.5	17.1	
Good (>52)	75.6	78.5	82.9	
**Country of birth**				72.0 (<0.0001)
Australia	94.6	95.0	91.1	
Other English speaking	3.6	2.6	4.1	
Europe	0.6	0.8	1.1	
Asia	0.7	1.0	2.7	
Other (incl. Middle East)	0.5	0.6	1.0	
**Area of residence**				420.3 (<0.0001)
Urban (major cities)	37.3	46.6	61.2	
Rural (inner regional)	36.5	32.4	26.5	
Remote (outer regional/ remote)	26.2	21.0	12.3	

^†^Achieved at Survey Five (age 31–36 years) (if missing, then from Survey Four) categorised as ‘Low’ - higher school certificate or lower (≤12 years), ‘Intermediate’ - trade/certificate/diploma or ‘High’ - degree/higher degree.

^‡^Sample sizes change slightly due to missing values for some variables.

*Mantel-Haenszel chi square used when >10% data was missing.

**Age at birth of first child is based on reported information up to Survey Five.

Compared to the other two education groups, a significantly smaller proportion of high educated women were separated/divorced/widowed, with the greatest proportion having never married (~89%). In contrast to high educated women, at baseline a larger proportion of low educated women were sedentary or had low physical activity levels; never/rarely drank or had risky drinking levels; were current smokers; had poor mental health; were Australian born; and lived in a rural or remote area.

While the above differences at baseline based on highest achieved education were noted, these covariates did not have a large effect on the association between education and body weight, as seen in the fully adjusted models (see Tables 2 &3, model 2).

## Discussion

This study investigated body weight trajectories over 13 years among Australian women aged 18–23 years at baseline. The results suggest that while the mean trend is increasing body weight for all women, adult education level is significantly associated with weight trajectory.

We found that high educated women benefited from gaining less weight over a 13 year period. This is consistent with other studies which have found an inverse association between education and long term weight gain [4–7]. A Finnish study [34], investigating the association between multiple measures of SEP and five year weight gain in mid-aged women, found that after full adjustment for a range of SEP measures (including parental education, childhood education, childhood and adulthood socioeconomic difficulties, own occupational social class and material resources) only the association between education and weight gain remained; suggesting this might be due to education preceding occupation and income [34]. Given this, one may assume that formal education itself encourages better health and a more promising weight trajectory; possibly through an increased knowledge of health behaviours and greater access to resources. However, assuming that knowledge results in positive behavioural change/practices should be questioned; as shown in a U.S. longitudinal study [6] which found the BMI trajectory of socially advantaged groups to be increasing and indeed higher than socially disadvantaged groups born 10 years earlier, although this could be confounded by timing of measurement.

While women with a high education at Survey Five had the lowest baseline weight and weight trajectory, two interesting findings were apparent regarding educational mobility. Firstly, women who remained with a low education at both time points had a steeper weight trajectory than those who went on to upgrade their education. Secondly, we found that lower educated women with the greatest educational mobility had a similarly favourable baseline weight and trajectory to those who had already achieved this high education earlier on. Our finding support those from a U.S. study of 18–30 year olds which found that, among white women, while education at baseline was only associated with BMI at this same time point, education at follow up (7 years after baseline) was associated with both baseline weight and weight at follow up [24]. Our analysis of educational mobility adds to this knowledge by highlighting that, with regards to baseline weight and weight trajectory, little additional advantage is seen in women who obtained this level of education early on compared to later.

This suggests that using education in early adulthood as a fixed marker of SEP may be inaccurate and in fact downplay the association between education and body weight trajectory. It may explain why some studies find an association between education and weight trajectory only among older women [13] or not at all when measuring education at baseline [15, 16]; while others, find a negative association when using education in later adulthood [8, 20].

Additionally, these results suggest that health behaviours/knowledge we might expect in highly educated women may be more related to factors operating earlier in life that lead to obesity, including early developmental patterns [35]. One explanation is that education attainment is influenced by IQ, however the early life environment in which cognitive ability and personality development are nurtured [36] must also be important, not least due to the types of resources available and psychosocial factors that make up that environment, as well as possible early socioeconomic disadvantage. We also tried conditioning for both mother’s and father’s education, separately and mutually, and found our associations remained the same, with marginally reduced estimates (results not shown). This suggests that even when we take into account early life SEP, there is still an effect of own education. It could be that some shared personality traits exist, which may make an individual more likely to engage in (and successfully obtain) a high education and also more successful at weight management, such as persistence and self-directedness [37].

The main strength of this study is having five waves of data collected over 13 years in a large sample. This gave women adequate time to have completed their education and allowed for a more in-depth analysis into changes in education level than would have been possible with fewer time points. Use of longitudinal methods accommodated for correlation between multiple observations per individual, while allowing for time-varying covariates and changes in behavioural/demographic characteristics which may influence body weight. Additionally, sensitivity analyses showed similar associations to those presented, including analyses using imputed data for both exposures and outcomes.

Potential study limitations should not be overlooked. Consistent with findings in other developed countries, a Melbourne study found an average weight gain of 0.4 kg/year [20]; while our study found an average gain in mean weight of 0.7 kg/year (8.6 kg over the follow-up period). While self-reporting includes the possibility of overestimated height and underestimated weight, both are found reasonable to use within epidemiological studies [38]. If weight underreporting is consistent, Baltrus et al. [13] suggest weight trajectory estimates should not be affected; although Brown et al. [5] suggest this may not apply to overweight/obese women, who have a greater tendency for weight underestimation, resulting in estimates biased towards the null. Since education is positively associated with height, we also looked at BMI trajectories and found the same associations as we did with body weight; we chose the latter as it offers a more interpretable result. Given that the significance of weight is dependent on height, we tried to account for this by adjusting all models for height centred at the cohort mean.

An overrepresentation of tertiary educated women in this cohort (12%, compared to 3% in the closes Australian census) [26], together with a slightly higher proportion of high educated women included (12%) than excluded (6%) from the sample may influence generalizability of results through selection bias.

## Conclusion

Our study highlights the importance of when education is measured, how it is used in analyses and the theoretical/causal model that is to be tested; all of which may influence the interpretation of results and the mechanisms through which SEP is thought to influence weight change. Using earlier education to measure the association between SEP and body weight trajectory may result in biased estimates, underestimating the association. High achieved education was significantly associated with a more favourable weight trajectory; with little increased advantage among those who had obtained this high education early on, compared to the women with a lower education who upgraded over the 13 year period. This suggests that behavioural characteristics and health knowledge often associated with a high education may already differentiate women early on; including personality traits related to weight management [37]; early life factors, such as food/flavour preferences [39]; and modelling of parental physical activity and nutritional patterns [40]. Overall, understanding the role of education and the mechanisms through which it may influence body weight may help to identify women, and hence children, at increased risk of an unhealthy weight trajectory.

## Electronic supplementary material

Additional file 1: **Inclusion/exclusion of subjects in our analyses, from women in the ALSWH cohort born 1973–1978.** (PDF 179 KB)

Additional file 2: **Baseline weight and weight gain** ^*^ **over 13 years by highest education** ^**†**^ in 1973–78 cohort ALSWH women, using multiply imputed data (n = 13 862**). ^*^random effects models (intercept only) with weight measured at age 18–23 years, 22–27 years, 25–30 years, 28–33 years and 31–36 years. ^†^education achieved at Survey Five (Low - higher school certificate or lower (≤12 years), Intermediate - trade/certificate/diploma, High - degree/higher degree). **women with at least one body weight. Model 1 – Baseline centred age, baseline centred height and area of residence. Model 2- Model 1 + country of birth, physical activity, alcohol intake, mental health, income management, self-rated health, age at first birth, living arrangements, marital status, shape dissatisfaction. (PDF 321 KB)

Additional file 3: **Baseline weight and weight gain** ^*^ **over 13 years by educational mobility** ^**†**^ in 1973–78 cohort ALSWH women, using multiply imputed data (n = 13 862**). ^*^random effects models (intercept only) with weight measured at age 18–23 years, 22–27 years, 25–30 years, 28–33 years and 31–36 years. ^†^change in education level from early-mid twenties to early-mid thirties: (Low - higher school certificate or lower (≤12 years), Intermediate - trade/certificate/diploma, High - degree/higher degree). Upgrade to high includes women who had a low (70%) or intermediate (30%) education who later upgraded to a high education. **women with at least one body weight. Model 1 – baseline centred age, baseline centred height and area of residence. Model 2 - Model 1 + physical activity, alcohol intake, mental health, self-rated health, number of children, smoking, age at first birth, living arrangement, marital status, health transition, shape dissatisfaction, income management and country of birth. (PDF 323 KB)
