# Supplementary material for: Educational mobility and weight gain over 13 years in a longitudinal study of young women
Source: BMC Public Health. 2014 Nov 25;14:1219. doi: 10.1186/1471-2458-14-1219 (PMC4289243; doi:10.1186/1471-2458-14-1219)
Supplement: Supplementary file 1 — Additional file 1: Inclusion/exclusion of subjects in our analyses, from women in the ALSWH cohort born 1973–1978. (PDF 179 KB) [file 12889_2014_7393_MOESM1_ESM.pdf]

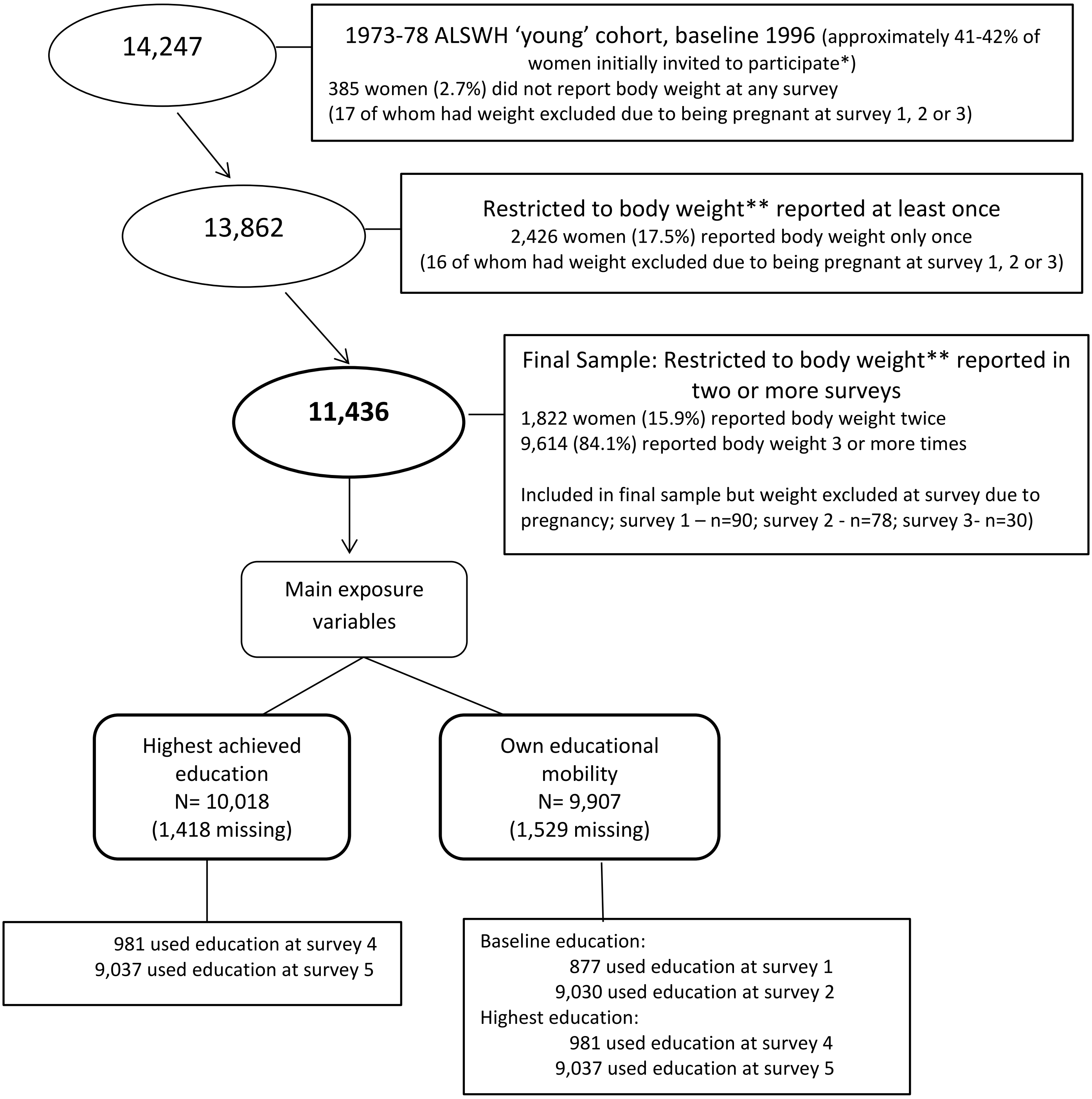

*see alswh.org.au/about/sample

**refers to non-pregnant body weight – reported body weight for women pregnant at survey 1, 2 or 3 was excluded (see methods)
