# Supplementary material for: Educational mobility and weight gain over 13 years in a longitudinal study of young women
Source: BMC Public Health. 2014 Nov 25;14:1219. doi: 10.1186/1471-2458-14-1219 (PMC4289243; doi:10.1186/1471-2458-14-1219)
Supplement: Supplementary file 2 — Additional file 2: Baseline weight and weight gain * over 13 years by highest education † in 1973–78 cohort ALSWH women, using multiply imputed data (n = 13 862**). *random effects models (intercept only) with weight measured at age 18–23 years, 22–27 years, 25–30 years, 28–33 years and 31–36 years. †education achieved at Survey Five (Low - higher school certificate or lower (≤12 years), Intermediate - trade/certificate/diploma, High - degree/higher degree). **women with at least one body weight. Model 1 – Baseline centred age, baseline centred height and area of residence. Model 2- Model 1 + country of birth, physical activity, alcohol intake, mental health, income management, self-rated health, age at first birth, living arrangements, marital status, shape dissatisfaction. (PDF 321 KB) [file 12889_2014_7393_MOESM2_ESM.pdf]

Additional File 2: Baseline weight and weight gain* over 13 years by highest education† in 1973-78 cohort ALSWH women, using multiply imputed data (n=13 862**)

	% weighted (unweighted)	Model 1 Estimate (95% CI)	Model 2 Estimate (95%CI)
**Baseline weight (kg)**		60.44 (59.99, 60.88)	57.58 (56.72, 58.44)
Difference in baseline weight by highest achieved education†			
High	49.1 (44.5)	Reference	Reference
Intermediate	29.7 (31.3)	2.07 (1.45, 2.69)	1.18 (0.59, 1.78)
Low	21.2 (24.2)	2.29 (1.53, 3.05)	1.11 (0.36, 1.87)
**Increase per survey (kg)**		0.72 (0.66, 0.77)	1.23 (1.16, 1.29)
Difference in increase per survey by highest achieved education†			
High		Reference	Reference
Intermediate		0.27 (0.23, 0.31)	0.24 (0.21, 0.28)
Low		0.34 (0.30, 0.38)	0.29 (0.25, 0.33)
Attenuation per survey (Survey*survey)		-0.01 (-0.02,-0.01)	-0.05 (-0.05, -0.04)

* random effects models (intercept only) with weight measured at age 18-23 years, 22-27 years, 25-30 years, 28-33 years and 31-36 years

† education achieved at Survey Five (Low - higher school certificate or lower (≤ 12 years), Intermediate - trade/certificate/diploma, High - degree/higher degree)

** women with at least one body weight

Model 1 – Baseline centred age, baseline centred height and area of residence

Model 2- Model 1 + country of birth, physical activity, alcohol intake, mental health, income management, self-rated health, age at first birth, living arrangements, marital status, shape dissatisfaction
