# Supplementary material for: Educational mobility and weight gain over 13 years in a longitudinal study of young women
Source: BMC Public Health. 2014 Nov 25;14:1219. doi: 10.1186/1471-2458-14-1219 (PMC4289243; doi:10.1186/1471-2458-14-1219)
Supplement: Supplementary file 3 — Additional file 3: Baseline weight and weight gain * over 13 years by educational mobility † in 1973–78 cohort ALSWH women, using multiply imputed data (n = 13 862**). *random effects models (intercept only) with weight measured at age 18–23 years, 22–27 years, 25–30 years, 28–33 years and 31–36 years. †change in education level from early-mid twenties to early-mid thirties: (Low - higher school certificate or lower (≤12 years), Intermediate - trade/certificate/diploma, High - degree/higher degree). Upgrade to high includes women who had a low (70%) or intermediate (30%) education who later upgraded to a high education. **women with at least one body weight. Model 1 – baseline centred age, baseline centred height and area of residence. Model 2 - Model 1 + physical activity, alcohol intake, mental health, self-rated health, number of children, smoking, age at first birth, living arrangement, marital status, health transition, shape dissatisfaction, income management and country of birth. (PDF 323 KB) [file 12889_2014_7393_MOESM3_ESM.pdf]

Additional File 3: Baseline weight and weight gain* over 13 years by educational mobility† in 1973-78 cohort ALSWH women, using multiply imputed data (n=13 862)**

	% weighted (unweighted)	Model 1 Estimate (95% CI)	Model 2 Estimate (95% CI)
**Baseline weight (kg)**		62.50 (61.80, 63.21)	56.21 (55.06, 57.37)
Difference in baseline weight by educational mobility†			
Stable low	20.7 (23.8)	Reference	Reference
Low-intermediate	10.4 (11.4)	-0.94 (-2.03, 0.15)	-0.84 (-1.85, 0.17)
Stable intermediate	20.0 (20.7)	0.53 (-0.38, 1.45)	0.74 (-0.14, 1.63)
Upgrade to high	13.3 (12.3)	-1.99 (-2.97, -1.02)	-1.19 (-2.14, -0.24)
Stable high	35.6 (31.9)	-2.02 (-2.77, -1.27)	-0.87 (-1.62, -0.12)
**Increase per survey (kg)**		1.07 (1.00, 1.13)	1.51 (1.44, 1.59)
Difference in increase per survey by educational mobility†			
Stable low		Reference	Reference
Low-intermediate		0.01 (-0.06, 0.06)	0.01 (-0.05, 0.07)
Stable intermediate		-0.12 (-0.17, -0.07)	-0.09 (-0.15, -0.04)
Upgrade to high		-0.28 (-0.34, -0.23)	-0.23 (-0.29, -0.17)
Stable high		-0.37 (-0.41, -0.32)	-0.32 (-0.37, -0.27)
Attenuation per survey (survey x survey)		-0.01 (-0.02, -0.01)	-0.05 (-0.05-0.04)

* random effects models (intercept only) with weight measured at age 18-23 years, 22-27 years, 25-30 years, 28-33 years and 31-36 years

† change in education level from early-mid twenties to early-mid thirties: (Low - higher school certificate or lower (≤ 12 years), Intermediate - trade/certificate/diploma, High - degree/higher degree). Upgrade to high includes women who had a low (70%) or intermediate (30%) education who later upgraded to a high education.

** women with at least one body weight

Model 1 – baseline centred age, baseline centred height and area of residence

Model 2 - Model 1 + physical activity, alcohol intake, mental health, self-rated health, number of children, smoking, age at first birth, living arrangement, marital status, health transition, shape dissatisfaction, income management and country of birth
